# Climate Conditions During a Rift Valley Fever Post-epizootic Period in Free State, South Africa, 2014–2019

**DOI:** 10.3389/fvets.2021.730424

**Published:** 2022-01-31

**Authors:** Assaf Anyamba, Richard Damoah, Alan Kemp, Jennifer L. Small, Melinda K. Rostal, Whitney Bagge, Claudia Cordel, Robert Brand, William B. Karesh, Janusz T. Paweska

**Affiliations:** ^1^Universities Space Research Association, Columbia, MD, United States; ^2^Biospheric Sciences Laboratory, NASA Goddard Space Flight Center, Greenbelt, MD, United States; ^3^Physics Department and Goddard Earth Sciences Technology and Research, Morgan State University, Baltimore, MD, United States; ^4^Center for Emerging and Zoonotic Diseases, Johannesburg, South Africa; ^5^Science Systems and Applications, Inc., Lanham, MD, United States; ^6^EcoHealth Alliance, New York, NY, United States; ^7^Execuvet, Bloemfontein, South Africa; ^8^Cuyahoga County Board of Health, Parma, OH, United States; ^9^Department of Soil, Crop and Climate Sciences, University of the Free State, Bloemfontein, South Africa

**Keywords:** climate variability, ENSO, post-epizootic, Rift Valley fever, mosquito vectors

## Abstract

Rift Valley fever virus (RVFV) activity in Southern Africa tends to occur during periods of sustained elevated rainfall, cooler than normal conditions, and abundant vegetation cover creating ideal conditions for the increase and propagation of populations of RVFV mosquito vectors. These climatic and ecological conditions are modulated by large-scale tropical-wide *El Niño*–Southern Oscillation (ENSO) phenomena. The aim of this 5-year study was to investigate climatic conditions during Rift Valley fever “post-epizootic” period in Free State province of the Republic of South Africa, which historically experienced the largest RVF outbreaks in this country. We collected satellite-derived rainfall, land surface temperature (LST), and normalized difference vegetation index (NDVI) data since 2014 to understand broad environmental conditions in the years following a period of sustained and widespread large RVF outbreaks (2008–2011) in the region. We found this post-epizootic/interepizootic period to be characterized by below-normal rainfall (~-500 mm), above LSTs (~+12°C), depressed NDVI (60% below normal), and severe drought as manifested particularly during the 2015–2016 growing season. Such conditions reduce the patchwork of appropriate habitats available for emergence of RVFV vectors and diminish chances of RVFV activity. However, the 2016–2017 growing season saw a marked return to somewhat wetter conditions without any reported RVFV transmission. In general, the aggregate vector collections during this 5-year period follow patterns observed in climate measurements. During the 2017–2018 growing season, late and seasonally above average rainfall resulted in a focal RVF outbreak in one location in the study region. This unanticipated event is an indicator of cryptic RVF activity during post-epizootic period and may be a harbinger of RVFV activity in the coming years.

## Introduction

Rift Valley fever (RVF) is an acute viral disease predominantly of domestic animals (cattle, buffalo, sheep, goats, and camels) and secondarily, of human populations. The endemic region of RVF covers most of sub-Saharan Africa, the Arabian Peninsula (Saudi Arabia and Yemen), and Madagascar (1, 2). Epicenters of epizootics and epidemics located in Eastern and Southern Africa are driven by persistent and above-normal rainfall associated with global scale *El Niño*-Southern Oscillation (ENSO) phenomena teleconnections (3, 4). Broadly, RVF outbreaks tend to occur in Eastern Africa during the positive phase of ENSO (*El Niño*) and in Southern Africa during the negative phase of ENSO (*La Niña*). The two phases describe the periods of persistent and above normal rainfall in each region leading to flooding of *pan/dambo* habitats. Flooding of these ecological niches where the various primary mosquito vectors of RVF-virus (RVFV) *Aedes* species and secondary *Culex* species emerge in massive numbers to trigger an outbreak. The impacts of an outbreak are varied and range from high rates of abortions and deaths in affected livestock to mild influenza-like illness and severe clinical symptoms in humans, including hemorrhagic manifestations, hepatitis, retinitis and encephalitis, and mortality in humans (~1–35%) to abortions and mortality in affected livestock (~80–100%) (1). The impacts on economies are pronounced, especially on livestock trade, and were estimated at $60M during the 2006–2007 outbreak in East Africa (5) and $12M (R203.4M) to the sheep farming sector alone during the 2010 outbreak in South Africa (6, 7). Due to its prominence as a cross-over pathogen, RVFV is listed as a biological agent by US government public health and defense agencies (Department of Defense, United States Department of Agriculture, Centers of Diseases Control and Prevention) and international public and animal health organizations (World Health Organization, Food Agricultural Organization, and the World Organization for Animal Health), requiring focused investigations in RVFV-endemic and neighboring regions.

One such investigation is *Understanding Rift Valley Fever in the Republic of South Africa* in which we are comprehensively studying an array of key facets influencing the RVF disease system using a One Health approach during the study period 2014–2019, which we refer to here as *post-epizootic* period. We interpreted this period to correspond to interepizootic/inter-epidemic period because of the likelihood of future RVF epizootics/epidemics. The One Health approach used is a collaborative, multisectoral, and transdisciplinary approach involving climate variable observations at regional level, vegetation, ecology and soil investigation, mosquito vector surveillance at local level, epidemiological investigations in livestock, wildlife, and human populations at farm level. The project therefore recognizes the interconnection between people, animals, plants, and their shared environment. The project is organized into eight work packages: 1. *Understanding the effects of climate and weather* (this study), 2. *Investigating vegetation ecology* (8), 3. *Investigating wetland soil properties* (9), 4. *Investigating ecological characteristics of RVFV vector mosquitoes*, 5. *Determining the seroprevalence of RVFV antibodies in farm workers* (10), 6. *Determining the seroprevalence of RVFV antibodies of farmed and free-ranging wild ruminants and domestic livestock* (11), 7. *Investigating changes in RVFV antibody levels in a sheep cohort*, and 8. *Comparison of cattle and buffalo serostatus in the Free State and Limpopo*. This paper reports on findings from *Understanding the Effects of Climate and Weather*, which has monitored and analyzed broad-scale satellite-derived climatic and environmental variables that influence RVFV mosquito vector populations. Among these variables are rainfall, considered the primary large-scale driver of RVFV activity, vegetation [normalized difference vegetation index (NDVI)], land surface temperature (LST), evapotranspiration, etc., which are proximate determinants of habitat conditions influencing survival and propagation of RVF vector populations (12–14). Climate variability characterized by year-to-year rainfall, vegetation, and land surface temperature are important broad scale drivers influencing the distribution in space and time of Rift Valley fever mosquito populations; therefore, understanding this component of the RVF disease system is critical to the implementation of various efforts to prevent, control, and mitigate potential outbreaks.

## Materials and Methods

### Study Area

**Figure 1:**
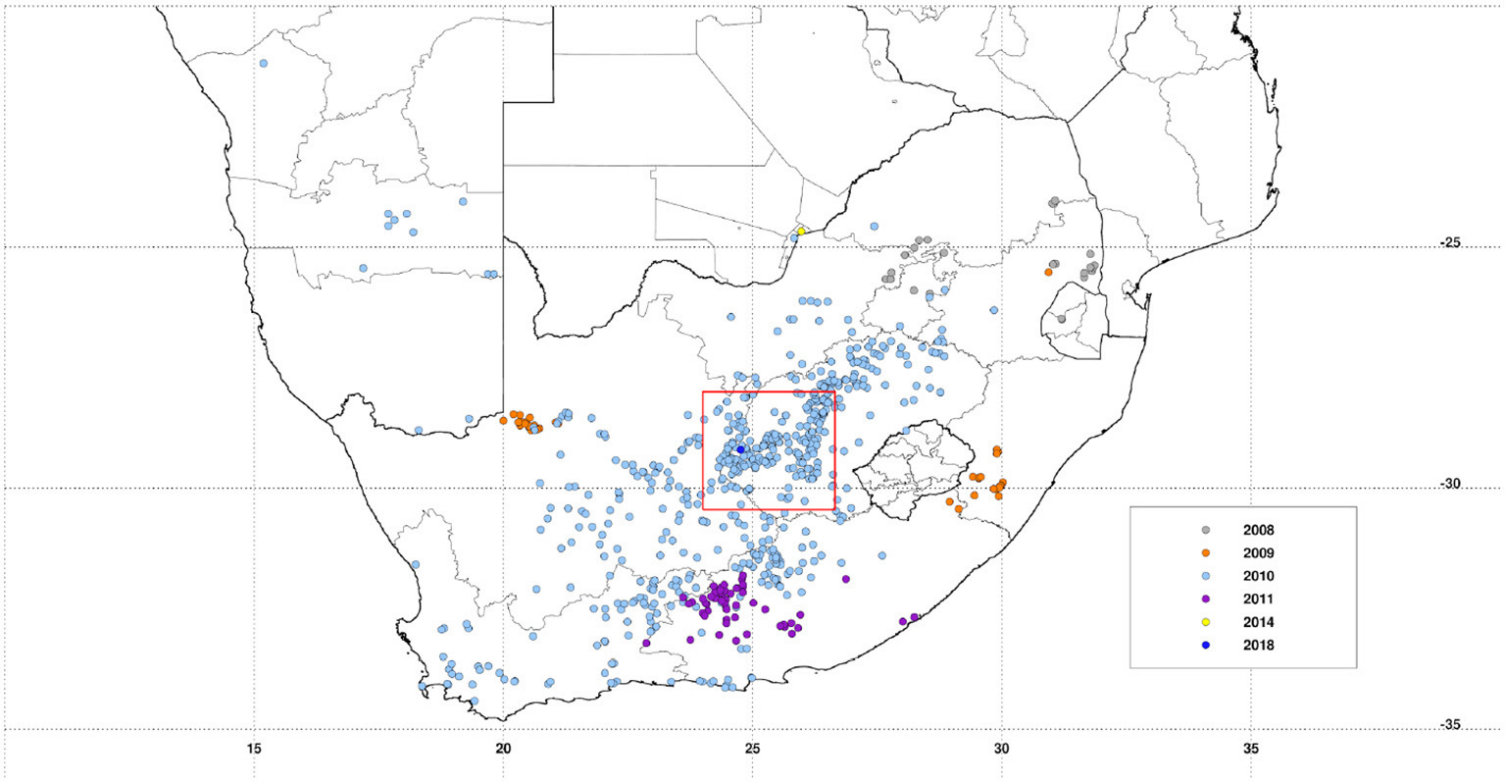
Distribution of recent Rift Valley fever activity (2008–2018) over the southern Africa region. *Rift Valley fever in the Republic of South Africa* study region is marked by a red square outline [28S−30.45S, 24E−26.65E], centered on Free State and Northern Cape Province border, the epicenter of multiple epizootics. Data plotted were derived from the World Organization for Animal Health (OIE) World Animal Health Information System (WAHIS) database.

**Figure 2:**
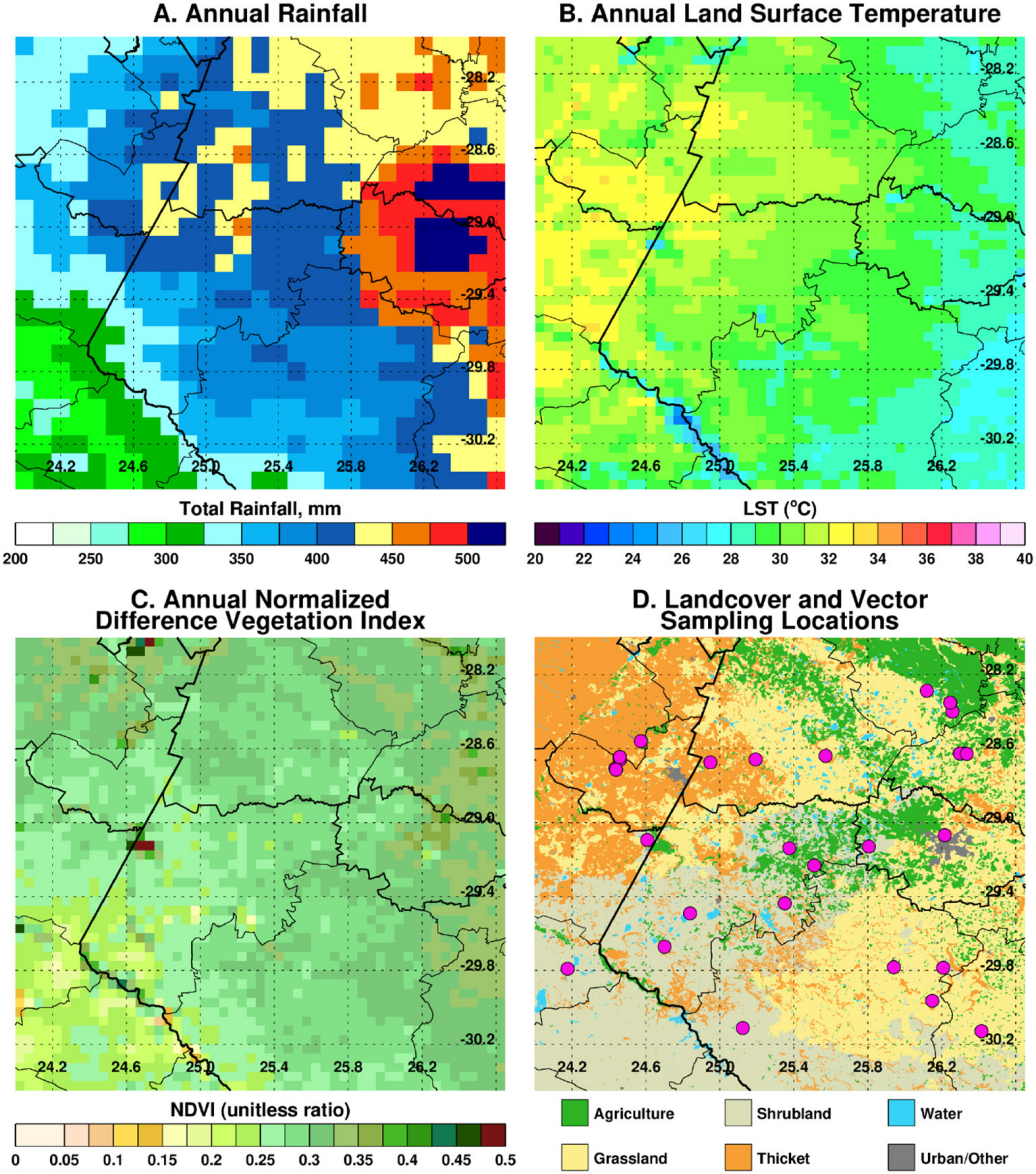
Climate metrics and land cover characteristic of the study region. In **(A)** annual long-term rainfall (1983–2019), **(B)** annual long-term land surface temperature (2000–2019), **(C)** annual long-term normalized vegetation index (2000–2019), and **(D)** land cover classification. The spatial variations and patterning in **(B–D)** to a large extent reflect the patterns in long-term rainfall.

The project is being conducted in a ~200 × 200 km area [28S−30.45S, 24E−26.65E] covering a large part of the Free State province and portions of both Eastern Cape and Northern Cape provinces. Significant epidemics were reported in these regions of South Africa in 1951, 1975, and 2010 with epizootics in 1951, 1975, 1984, 1999, 2008, and 2009, 2010, and 2011 (15, 16) with apparently quiescent inter-epidemic periods. Many of these epidemics had their epicenter in the Free State as can be observed from the recent epizootics as shown in Figure 1. Annual long-term rainfall in the region ranges between ~200 mm to the west and southwest of the region and a maximum of ~550 mm to the eastern and northeastern parts of the region. The climatological spatial patterns of land surface temperature with maximum values of ~35°C in the west/southwest, and normalized difference vegetation index with maximum values of ~0.45 in the east/northeastern parts of the region reflect the long-term annual mean patterns of rainfall. The combination of these climate metrics with the underlying geology has over time produced landcover patterns dominated by grasslands, savanna, and Nama-Karoo biomes (17), which includes fynbos elements, shrubs, and woodland species. Embedded in these three biomes are the azonal wetlands, which include the study area pan habitats, with vegetation distinct from the surrounding upland vegetation (8). Dryland agriculture that is heavily dependent on variable rainfall is practiced in the east, while locations in the drier west and southwest use irrigated agriculture to buffer against low and variable rainfall. The study area receives on average of ~96% of rainfall between September and May and 4% between June and August, considering the southern hemisphere summer rainfall season. There is, however, high interannual variability in rainfall producing periods of above normal rainfall and floods and episodes of very low rainfall and extreme drought. Periods of above-normal rainfall like 2009–2011 create conditions for outbreaks to occur and propagate. The study region thus has an approximately east–west ecological gradient from Bloemfontein to Mokala National Park (MNP) that can be observed in climate metrics and land cover patterns (Figure 2).

### Data

#### Climate Data

Three satellite-derived climate data sets are used in evaluating the patterns of rainfall and land surface conditions over the region during the study period (2014–2019). The three data sets are (a) daily/monthly rainfall from the Africa Rainfall Climatology (ARC) data, (b) monthly normalized difference vegetation index, and (c) monthly land surface temperature. Details on these datasets are given below:

(a) African Rainfall Climatology (ARC) dataset is sourced from the National Oceanic and Atmospheric Administration (NOAA)—Climate Prediction Center (CPC) archives. ARC data are derived from several satellites and *in situ* sources, including the polar orbiting Special Sensor Microwave/Imager and Advanced Microwave Sounding Unit microwave sensors, infrared bands of the geostationary METEOSAT platforms, and rain gauge measurements from the Global Telecommunications System daily total rainfall product. The data are mapped to a spatial resolution of 0.1° × 0.1° over Africa and the Middle East. These data are available as a daily time series from 1983 to present (18).
(b) Normalized difference vegetation index (NDVI) data are derived from NASA's Earth Observing System Moderate Resolution Imaging Spectroradiometer (MODIS) instrument aboard the Terra (EOS AM-1) spacecraft. The NDVI and similar vegetation indices are widely used to infer the photosynthetic capacity of vegetation and are used as a land surface input in various weather, climate, biogeochemical, and hydrological models (19). Applications of normalized difference vegetation index are numerous and varied and include agricultural monitoring, famine early warning, ecological monitoring for habitats indicative of pest and arthropod vector emergence and survival, and determination of land use and land cover changes, among others (12, 20–22). The normalized difference vegetation index is simply the ratio of the difference between the near-infrared and red reflectance to their sum; since green leaves with dense chlorophyll are more reflective in the near-infrared wavelengths than in the visible, this ratio is higher (approaching one) for healthy green vegetation and lower (approaching zero) for stressed vegetation (23). MODIS normalized difference vegetation index data are derived from the red and near-infrared bands, centered at 648 nm and 848 nm, respectively. The reflectance data are atmospherically corrected and masked for cloud, cloud shadow, and aerosol contamination (24). In this study we use the global monthly Climate Modeling Grid (CMG) MOD13C2 product with a spatial resolution of 0.05° × 0.05° (~5.5 × 5.5 km) aggregated from nominal 250 m MODIS NDVI.
(c) Land surface temperature (LST) was also derived from the MODIS instrument. Land surface temperature is a key parameter in land surface processes affecting climate and therefore influencing the biology, organisms, and ecosystems from local to global scales. Changes in land surface temperatures can induce convection at the boundary layer and influence air temperature, surface winds, cloudiness, and precipitation (25). All these variables influence habitat conditions of mosquito vectors. Land surface temperature has proved useful for agricultural applications in estimating crop water demands and drought severity assessments (26). It is also an emerging variable in vector-borne disease applications (21). We used land surface temperature to infer temperature conditions on the land surface especially in vegetated areas, which serve as potential vector emergence sites during the study period. In this study, we use the global Climate Modeling Grid (CMG) product MOD11C3 at 0.05° spatial resolution. This data set is derived from daytime and nighttime thermal infrared measurements in bands 31 (10.8–11.3 nm) and 32 (11.8–12.3 nm) using the day/night land surface temperature algorithm. Cloud screening is performed using the MODIS cloud mask product (MOD35_L2), prior to the land surface temperature calculation.

#### Mosquito Vector Data

To complement the satellite-based climate observations, adult floodwater mosquito vectors were sampled by the vector ecology team at over 21 locations daily (shown in Supplementary Figure 1). Given the sampling strategy this amounts to every 2 weeks per site during the growing season (September–May). Sampling was performed using US Centers for Disease Control and Prevention (CDC) CO_2_-baited traps placed at dusk to lure feeding adult female mosquitoes from diurnal resting locations. The sampling schedule was designed to be flexible to allow sampling all sites within the week. However, due to various issues at project startup, there was no regular sampling until the 2015–2016 season. In addition, significant weather events at times, including excessive rainfall or severe winds, made it impossible to access some sites and set traps, forcing the team to trap at alternate sites. In this paper we use aggregated adult female mosquito population collection numbers sampled during the project period to compare to the 5-year variability in climate conditions. The vector data presented in this paper are adjusted for trap effort. Trap effort was summarized as the number of hours the trap was open multiplied by the number of traps that were set. For instances where the time the trap was open was not available, the median of all available data was used. Number of traps was available for all data. Thus, the adult female mosquito numbers were divided by this trap effort (hours trap was open multiplied by number of trap sets) for summary numbers of adult female mosquitoes, and this trap effort was included as an offset in modeling the adult female mosquito number.

### Data Analysis

#### Satellite Data Treatment

We processed, mapped, and subset all satellite rainfall, LST, and NDVI data within the study region extent (28S−30.45S, 24E−26.65E). For each climate variable, we computed both daily and monthly long-term means and corresponding absolute and standardized anomalies. We also examined the growing/rainfall season conditions by calculating seasonal anomalies. For the growing season we divided the season into early (September–November; SON), peak (December–February; DJF), and end (March–May; MAM) to examine the evolution of growing conditions, classified as below-normal, normal, and above-normal. For given vector sampling locations we tracked the seasonal growing conditions of the location using the seasonal rainfall cumulative metric by comparing the daily cumulative rainfall against the daily long-term mean. Daily cumulative rainfall values above the daily long-term mean values are a proxy for potential flooding of dambos/pans and therefore conducive to the emergence and propagation of vectors in general, but RVFV vectors in particular (27, 28). In all cases, at monthly or seasonal time scale we have calculated anomalies using two complementary methods as:

(a) Absolute anomalies *x*′ = *x* − *x*
(b) Standardized anomalies $z=\frac{x-\underline{x}}{s_{x}}=\frac{x^{{}^{\prime }}}{s_{x}}$

**Figure 3:**
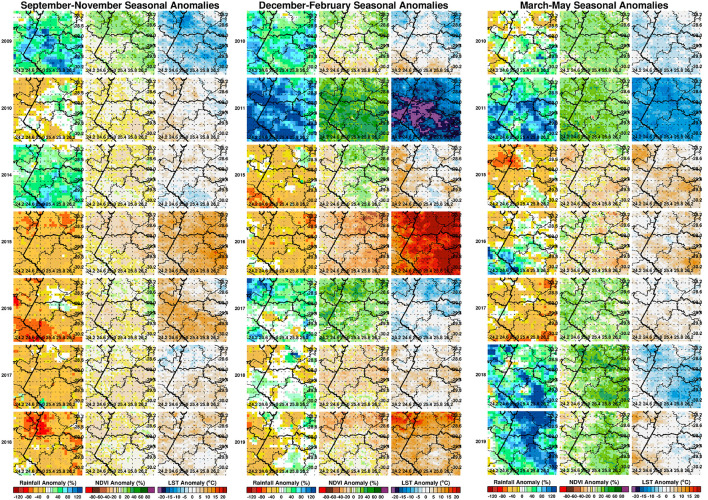
Seasonal anomaly patterns of rainfall, normalized difference vegetation index, and land surface temperatures for epizootic seasons (2009/2010, 2010/2011) and study post-epizootic/interepizootic period seasons from 2014–2019. Seasons are defined by early (September–November: SON), mid (December–February: DJF), and end (March–May: MAM). Rainfall and normalized difference vegetation index anomalies are expressed as percentage departures while land surface temperatures are expressed as absolute departures from the respective seasonal long-term means.

Where *x*′ is the anomaly for a given month (e.g., January) or seasonal anomaly (DJF), *x* the absolute values of a given month or season, the respective long term means or climatology values of the respective month and season, *z* the standardized anomaly or *z*-scores for a given month or season, and *s*_x_ the corresponding standard deviation. The effect of standardization is to remove influences of local variability so we can compare the difference over space and over time from the different climate measurements (29). Results of absolute anomalies are presented in Figure 3 and the standardized anomalies are given in the Supplementary Figure 2. For ease of interpretation by the reader, we have expressed absolute anomalies as percentage departures from the long-term mean for rainfall and normalized difference vegetation index metrics. Anomalies during the epizootic/period (2009–2010, 2010–2011) are included for reference purposes representing the most recent epidemic/epizootic period.

#### Vector and Climate Data Analysis

To investigate the relationship between vector populations and climate/environmental data, we employed a negative binomial regression model of the form,


$$\begin{array}{l} M_{\text{f}}=\text{Rainfall}+\text{NDVI}+\text{LST}+\text{Offset} \end{array}$$

where *M*_f_ is the number of adult female mosquitoes and Offset is the number of hours the traps were open (or median) ^*^ number of traps, to compare monthly rainfall, monthly normalized difference vegetation index, and monthly land surface temperature with total adult female vector populations sampled over the entire region. As the outcome measure, number of adult female mosquitoes, is a count variable, we employed a negative binomial regression approach with an offset for trap effort. The model structure maintains the data as count data with the negative binomial approach, and inclusion of the offset accounts for varying “rates” of mosquito capture—instances in the field data collection where number of traps or hours the trap was open might have varied from collection to collection based on field conditions—which would impact the number of mosquitoes caught at each collection time point. The offset employed in this model is a composite of both how long the trap was open and the number of traps that were set. For records in which the number of hours the trap was open was not present, we took the median of available data and multiplied that by the number of traps that were open during that collection time point. Complete data were available for number of traps set for all data collection time points.

## Results

### Spatial and Temporal Patterns of Climate Anomalies

**Figure 4:**
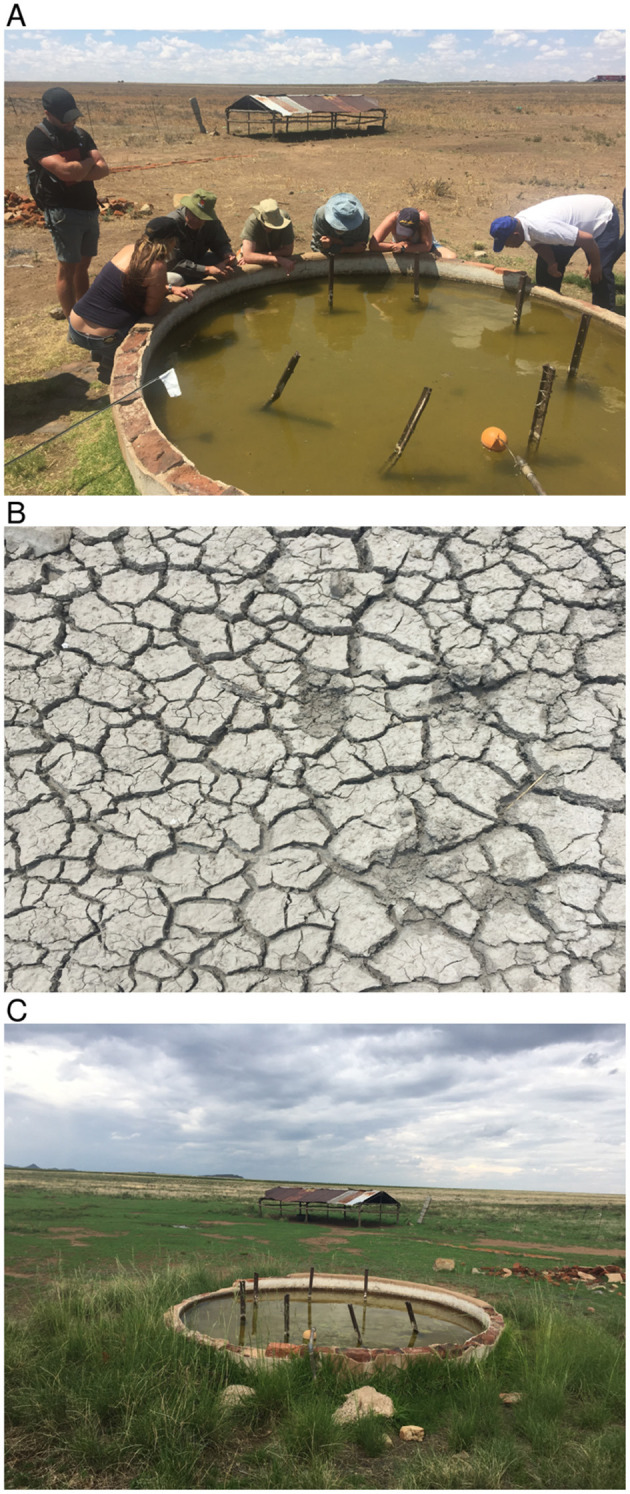
Contrasting conditions in and around Meadows pan habitat (location shown in Supplementary Figure 1). [**(A)**-top] severe drought conditions in March 2016 at the peak of the growing season (rainfall total: ~75.82 mm, long-term mean: 109.65 mm; normalized difference vegetation index: ~0.28, long-term mean: 0.44; land surface temperature: 34.6°C, long-term mean: 31.9°C), [**(B)**-middle], desiccated dambo/plan floor during the same time period, and [**(C)**-bottom] March 2018: vegetation growth after sustained wet conditions (rainfall total: ~182.80 mm, long-term mean: 109.65 mm; normalized difference vegetation index: ~0.51, long-term mean: 0.44; land surface temperature: 28.04°C, long-term mean: 31.9°C).

We first examined the spatial patterns in climate variable anomalies. In order to reduce the amount of data to examine we show the patterns by season: early season (September–November), mid-season (December–February), and end season (March–May). Geographic patterns of absolute anomalies for the study area are presented in Figure 3 (see standardized anomalies in Supplementary Figure 2 for reference). Anomalies during 2009–2010 and 2010–2011 are included for reference purposes, representing the recent epidemic/epizootic period. The patterns show that the epizootic period was dominated by an early start (2009–2010) to the season with favorable conditions for RVFV vectors during SON 2009 (widespread rainfall, above normal vegetation conditions and cooler than normal land surface temperature) that propagated into the mid-season (DJF) and further enhancement of these conditions in the last quarter of the season (MAM) for both vegetation and land surface temperature. The 2010–2011 epizootic period had a delayed start of the season but the rest of the season from December 2010 to May 2011 had enhanced and favorable conditions for the emergence of RVFV vectors and subsequent outbreaks as shown in Figure 1. During the study period 2014–2019, classified as post-epizootic/interepizootic period, the study region has been dominated by below normal rainfall, drier than normal vegetation conditions, and above normal land surface temperatures. The exceptions to this general pattern are: early-season during the 2014–15 season (SON2014), mid-season (DJF) in the 2016-2017 season, and late seasons in 2017–2018 (MAM2018) and 2018–2019 (MAM2019). The drier than normal conditions during this period reached a record low during the mid-season (DJF) 2015–2016 period with rainfall 60–80% below normal, normalized difference vegetation index ~80% below normal, and land surface temperatures up to +20°C above normal over most of region (Figure 3). The contrasting patterns of land surface conditions during this post-epizootic period are best illustrated by field work evidence as shown in Figure 4. In drought years as shown in Figures 4A,B, due to excessive livestock herbivory, there is no vegetation cover outside wetlands, and even in certain pan habitats, all wetlands vegetation, which is generally considered impalatable, is totally grazed, which would result in NDVI of 0 or near 0 representing bare soil or scant vegetation cover. The amount of vegetation cover—density, and the type of vegetation—wetland species adapted to anaerobic conditions, are important as habitat for mosquito vectors. While NDVI is useful when correlated with rainfall, it is of specific importance for the mosquito vectors as they require wetland vegetation which, in the pans and palustrine habitats in the Free State, is embedded in the surrounding vegetation, and far more limited in extent. Because wetland vegetation is so limited in size in the Free State, green up in vegetation surrounding pan habitats is prominently detected from background by satellite measurements of vegetation photosynthetic capacity represented as high NDVI. Figure 4C illustrates this with the recovery and vegetation growth after sustained wet conditions in March 2018 during the 2017–2018 growing season (Figure 3: March–May 2018).

**Figure 5:**
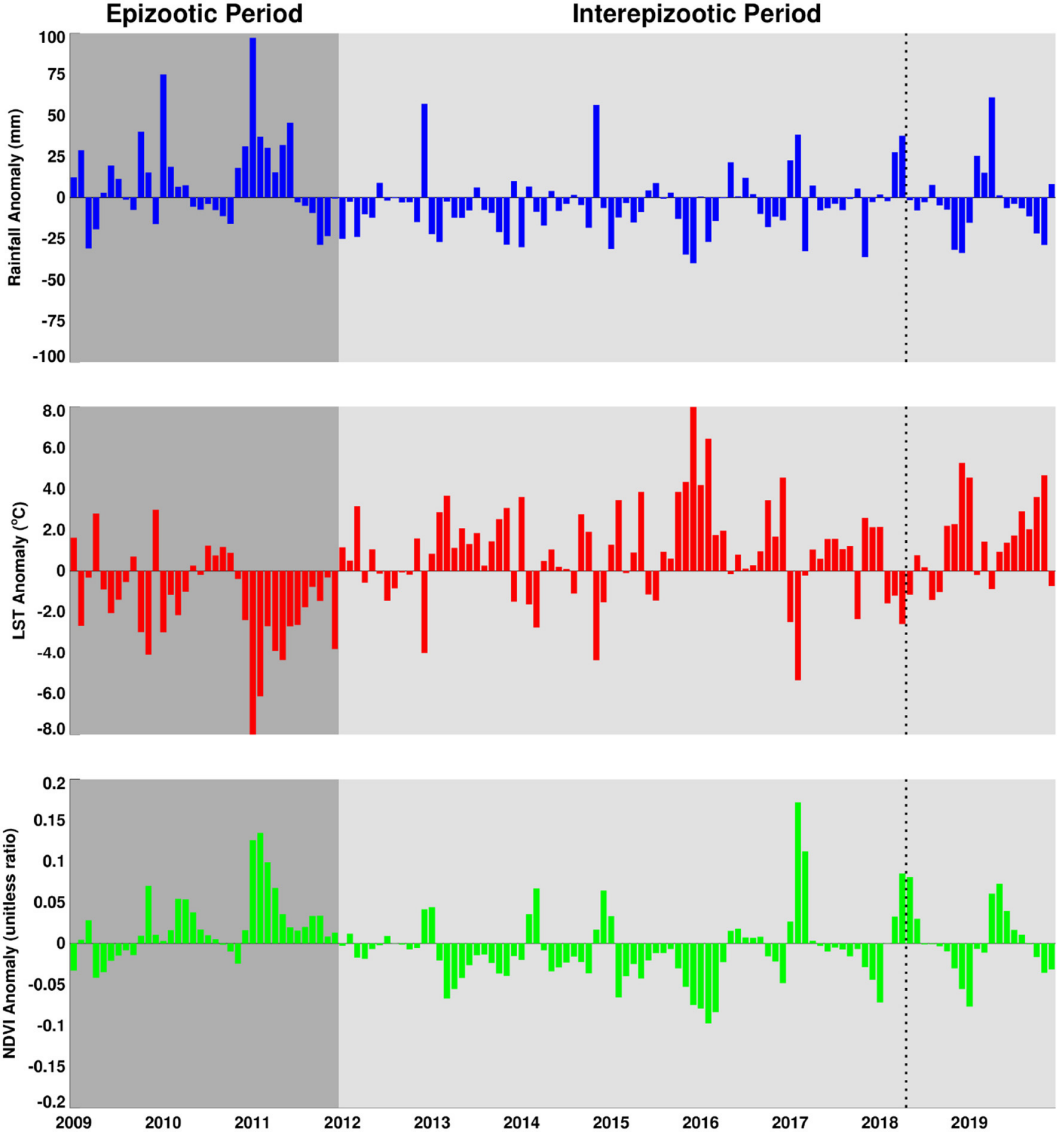
Anomaly time series of rainfall, land surface temperature and normalized difference vegetation index for the period 2009–2019. The Rift Valley fever epizootic period (2009–2011) is shaded in dark grey and shows predominantly above normal rainfall, lower than normal land surface temperatures, and above normalized difference vegetation index and opposed to the interepizootic/post-epizootic period (2012–2019) with lower than normal rainfall and normalized difference vegetation index and persistence of higher than normal land surface temperatures marked by the extreme drought of 2015–2016. The dotted line shows the approximate timing of the isolated outbreak during 2017–2018 growing season.

The area averaged climate anomaly time series for the region shown in Figure 5 illustrate that the post-epizootic period 2012–2019 (study period starts in 2014) has been dominated by below normal rainfall, above normal land surface temperatures, and below normal vegetation conditions. This is opposed to the epizootic period 2009–2011 which was characterized by above normal rainfall, below normal land surface temperatures, and above normal vegetation conditions. This figure also illustrates direct correlation between rainfall and NDVI but an inverse relationship between these two parameters and LST. It is clear that during the epizootic period (2009–2011), the intensity of the amplitudes and the spread of the base months starting early 2009 until later 2011 they are ~18/23 months of above normal rainfall/NDVI and the inverse LST. When compared with successive periods in the following years, this pattern is not persistent enough in magnitude nor of the temporal range required for a large scale outbreak.

**Figure 6:**
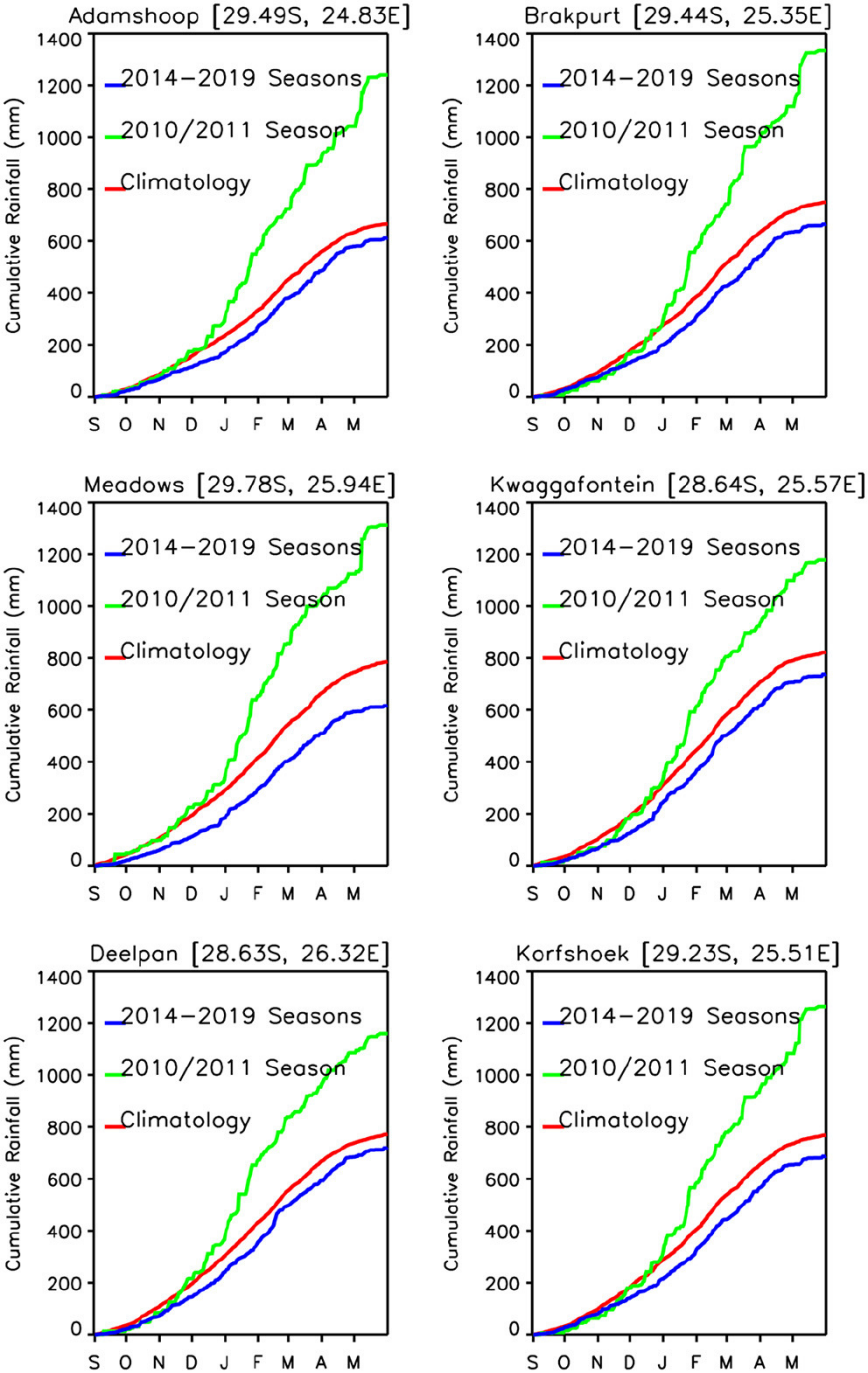
Daily cumulative rainfall time series trajectories for epizootic period (2010/2011; green) and interepizootic period (average of 2014–2019; blue) compared to the daily long-term mean rainfall (red) for six selected study sites. The epizootic season (2010–2011) shown in green, was above the long-term mean, with a rainfall excess ranging between ~200 and ~600 mm across study sites by May. The study post-epizootic/interepizootic period shows persistently below normal rainfall with a shortfall of ~100 mm by May across all study sites.

The area average growing season absolute rainfall for two seasons (2009/2010, 2010/2011) was 50.80 mm and for the 5 years of the study period was 39.4 mm. In terms of growing season cumulative rainfall anomalies, the epizootic period had excess rainfall on the order of ~+367.50 mm compared to a deficit of ~-266.25 mm for the entire post-epizootic growing seasons period (2012–2019). Supplementary Table 1 shows the study area's average metrics for rainfall, normalized difference vegetation index, and land surface temperature for the entire period from 2012–2019. Examining cumulative rainfall trajectories for six selected study sites (Supplementary Figure 3), we find that only 2016/2017 and 2017/2018 are near normal and slightly above normal rainfall toward the end of the season. Other than that, none of the growing seasons during the study period exhibited persistent above normal rainfall that was sufficient enough to create ideal conditions to trigger an outbreak as was the case during the epizootic period in 2010/2011. In totality, the daily cumulative rainfall trajectories for the selected study sites indicate that there is a clear and contrasting difference between the epizootic (2010/2011) and study/post epizootic period (2014–2019) rainfall conditions (Figure 6) that is also reflected in other climate metrics.

### Implications for Vector Populations

**Figure 7:**
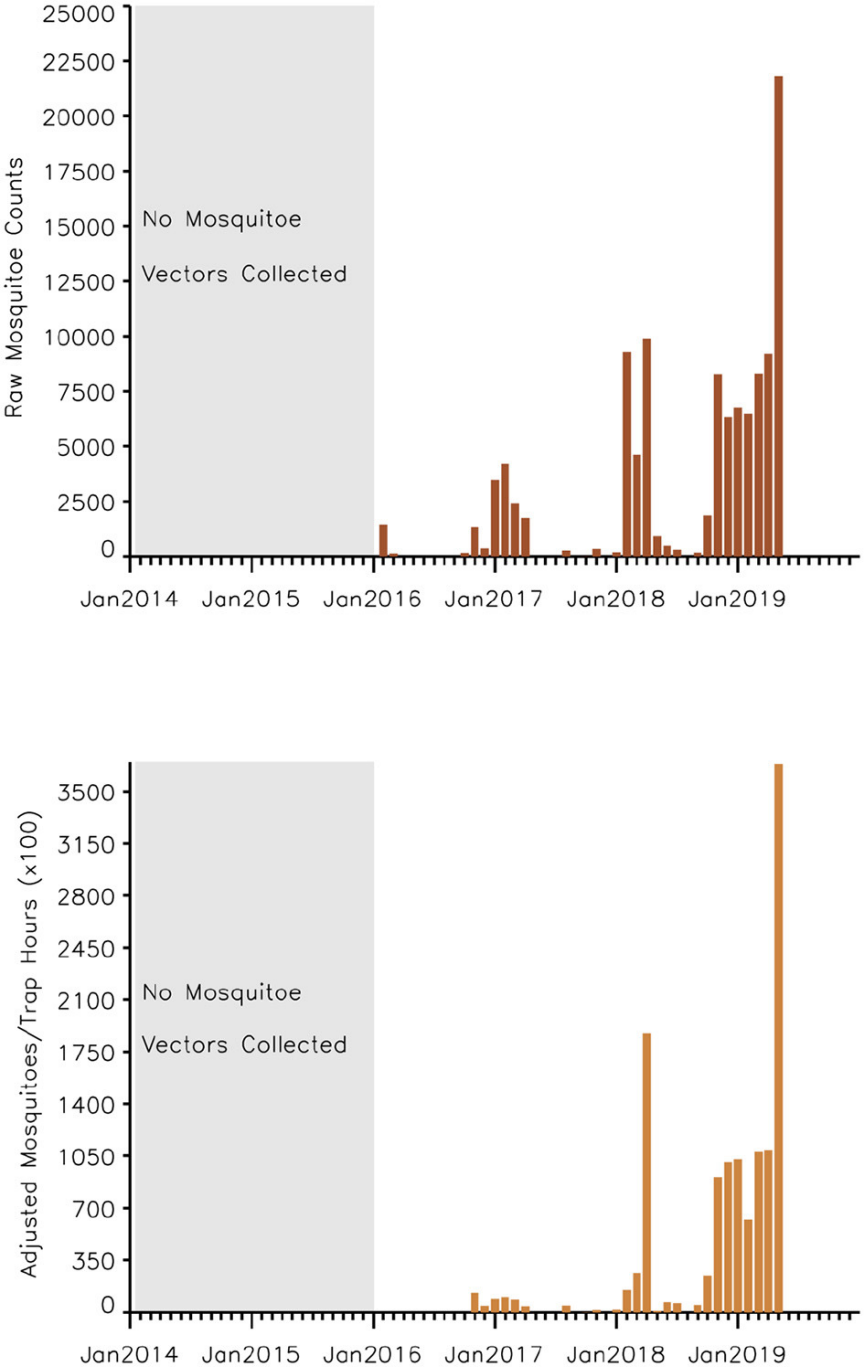
Time series of aggregate mosquito vector counts (of various floodwater *Aedes spp*. and *Culex spp*.) for the study period September 2014–2019 (**top**) raw mosquito vector counts (**bottom**), adjusted mosquito vector counts accounting for trap effort during the study period. The number of vectors collected shows an increasing trend over time in concert in the increase in rainfall improving habitat conditions. Taking into consideration the trapping effort, 2014–2015 and 2015–2016 seasons had no vectors collected during this period of severe drought conditions.

**Figure 8:**
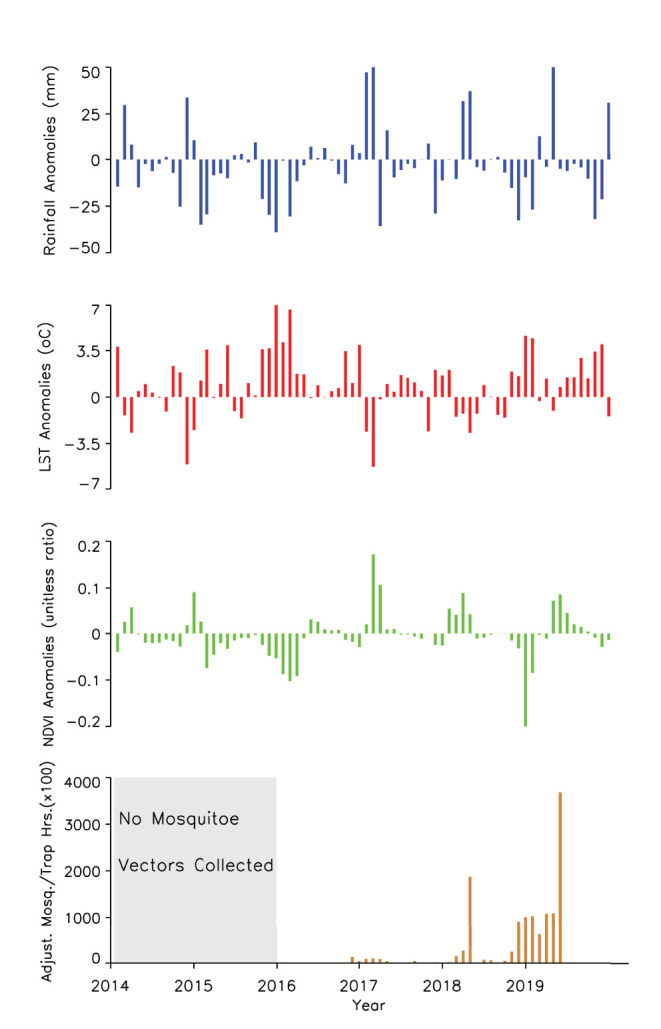
Time series measurements of rainfall, land surface temperatures, normalized difference vegetation index anomalies, and adjusted mosquito vector collections during the study period. Vector collections only became prominent when rainfall and NDVI were trending toward above normal during the growing seasons from 2017–2019.

**Figure 9:**
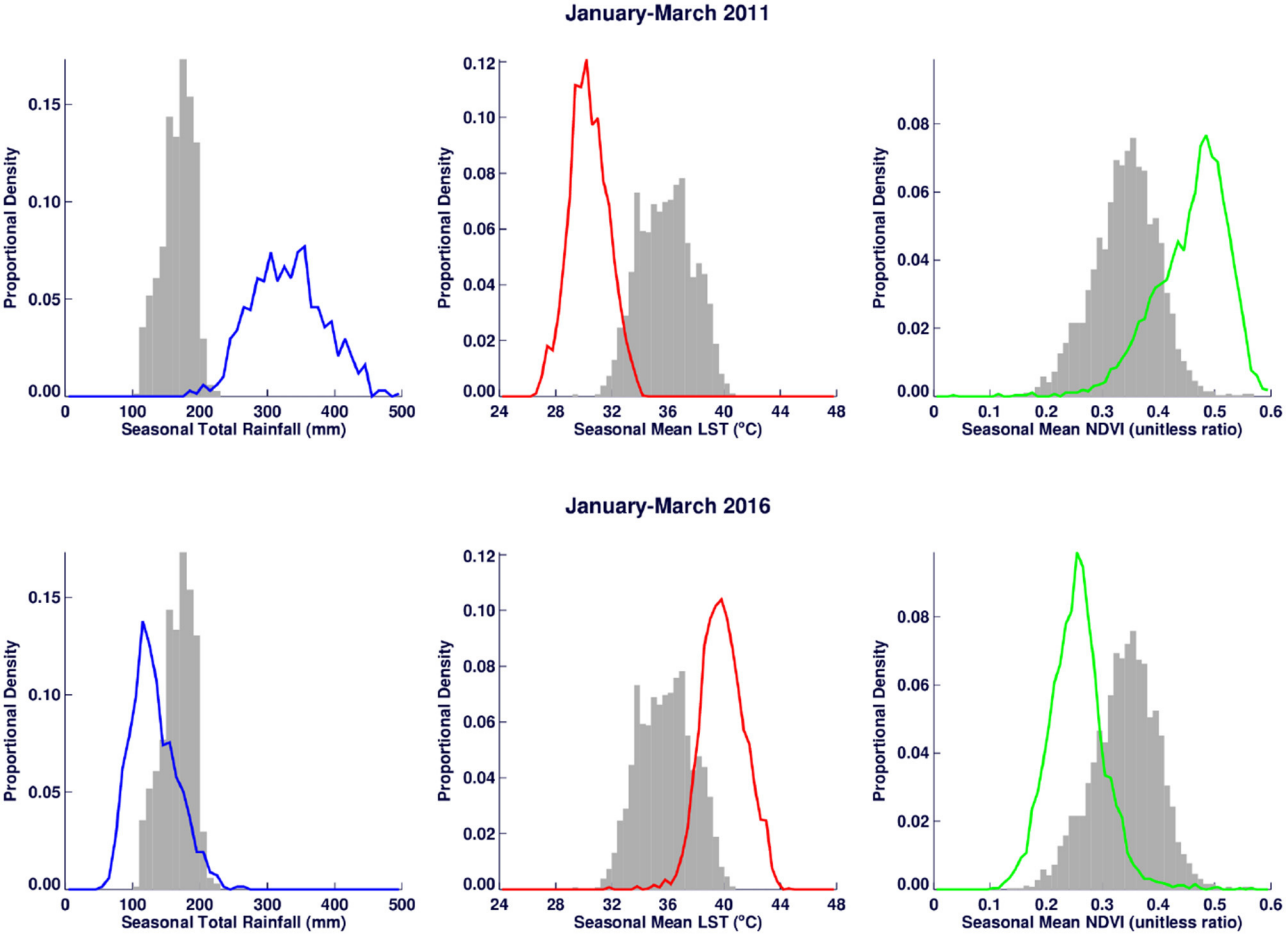
Climate variable distribution comparisons between Rift Valley fever epizootic period (January–March 2011) (**top**) and post-epizootic period (January–March 2016) illustrates clear shift to the right of the long-term mean distribution (in rainfall and normalized difference vegetation index) and to left (land surface temperature) during the epizootic period. The distribution shifts are reversed during the interepizootic period. The gray bars represent the long-term mean distribution for each variable.

A time series of the total monthly mosquito vectors collected and the corrected for trap effort during the study period are shown in Figure 7 and indicate an increasing trend over the 5-year period. Low numbers of vector populations were collected during the 2016–2017 seasons, with the lowest/none during the 2015–2016 period after adjustment for trap effort during the great desiccation period. However, since the 2016/2017 growing season there has been an increase in the number of vectors collected with the highest adjusted numbers during the 2018–2019 growing season. These patterns mirror the trends in climate variables shown in Figures 5, 8 especially for rainfall and as reflected in spatial patterns in Figure 3. A negative binomial regression analysis of climate variables averaged for the entire study region (rainfall, normalized difference vegetation index, land surface temperature as independent variables) and vector populations (dependent variable) for all growing seasons under study shows that, at the monthly time scale, both NDVI and LST are significantly positively correlated with vector populations, while rainfall is negatively, and not significantly, correlated (Table 1, adjusted R-squared 0.273). NDVI, LST and Rainfall were not collinear. To assess whether there was any seasonal time trend in the data, we incorporated a smooth function for season into the model of mosquito abundance using the mgcv package in R statistical software and compared the two models by AIC. The model without the smooth for season had the lower AIC and is thus the model we chose. Interestingly, the estimate for NDVI was substantially higher than the others in the model, indicating a strong positive relationship between NDVI and number of adult female mosquitoes. Since NDVI is a linear function of rainfall in semi-arid areas like the study area (30), it captures the memory of previous and present rainfall events including all surface conditions. It has been shown that environmental temperatures of 25°C−30°C are ideal for the propagation of Rift Valley fever and other disease vectors (31, 32). This is also reflected in land surface temperature shifts during the Rift Valley fever outbreaks (21). As can be noted in Figure 9 as an example, comparing the aggregate climate variable conditions for land surface temperature between the epizootic (2011 January–March) and the interepizootic (2016 January–March) periods, temperature distribution shifts leftwards to ~26°C–~33°C during the epizootic period, while during the post-epizootic/interepizootic period, the distribution shifts right of climatology to ~36°C−44°C. We performed a *t*-test to determine the significance of the differences in means between the peak seasons (January–March) of the two representative years for epizootic period against the interepizootic year (null hypothesis H0: means are the same, alternative HA: the means are different) for all the climate variables. Results indicate that we reject the null hypothesis, as indeed the means differ significantly between epizootic (2010, 2011) and post-epizootic/interepizootic year (2016) at 95% confidence level. The former conditions favor the emergence and propagation of large populations of Rift Valley fever vectors. Supplementary Figure 4 also illustrates that for the entire study region, rainfall is consistently below or near the long-team mean during this post-epizootic period unlike the above normal rainfall conditions during the epizootic period. In addition, peak rainfall is shifted later into the season and has two peaks in February and April during the interepizootic period, which differs from the peak of January for both the climatology and the epizootic period. Accordingly, the normalized difference vegetation index is consistently below normal during the post-epizootic/interepizootic period and only approaches the long-term mean toward the end of the season in concert with rainfall. Land surface temperatures are correspondingly consistently above the long-term mean for most of the entire growing season, conditions only reach below 30°C in April and May, and it is therefore no surprise that the bulk of vectors collected throughout the entire study period are in May (Supplementary Figure 5). This shift in the combined climate and ecological conditions may explain the cryptic and localized outbreak that occurred during this interepizootic period in the southwest corner of the study region in May 2018 (Figures 1, 5) (33, 35).

**Table 1:** Regression results of female adult vector populations and rainfall, normalized difference vegetation index (NDVI), and land surface temperature (LST).

**Variable**	**Estimate**	**Std. Error**	***p*-value**
Rainfall	−0.00281	0.01419	0.843
NDVI	12.55334	5.53645	0.023^*^
LST	0.14202	0.05395	0.008^*^

* *indicates a statistically significant result at the 0.05 level*.

*Adjusted R-squared: 0.273*.

## Summary and Conclusions

The post-epizootic period has been characterized to a large extent by below normal rainfall, poor vegetation conditions, and above normal land surface temperature during the growing/rainfall season (September–May). These conditions are dramatically exemplified by the nadir during the 2015/2016 growing season with low rainfall, depressed vegetation conditions, and abnormally high land surface temperatures. For the entire study period, rainfall and normalized difference vegetation index have peaked later in the season in April; a month or two later than average. The conditions have implications for vector abundance both through space and time: a small population of vectors was collected in 2014–2016/17 seasons; only until later in the study period have we had an increased number of collections. Also given that conditions have been peaking later in the season, thermal conditions have not been favorable for propagation of large numbers of vectors with early and mid-season land surface temperatures measuring above 30°C. This aspect may partly account for the localized outbreak in April 2018 late in the growing season. As a whole; during the post-epizootic period, we can conclude that conditions have not been favorable for large scale regional Rift Valley fever activity.

Field observations have also shown us that the Free State region is a complex landscape, with numerous potential habitats—land of 10 000 pans (34)—both natural and artificial. In this respect, large-scale monitoring of drivers of climate variability such as ENSO and monitoring of proximate regional environmental indicators (rainfall, NDVI, soil moisture, etc.) to detect specific shifts in patterns can support targeted vector surveillance in high-risk areas and concurrent vaccination campaigns. This will be an effective method to prevent and control RVF and minimize the scale of costs and damage such as those during and after the 2009–2011 epizootic period. Under large-scale flood conditions, it would be impossible to manage or control an outbreak; most farmers will not be reached due to unnavigable road networks. Given the critical importance of agriculture and livestock farming, in particular to South Africa's economy and to rural livelihoods, it is imperative that the livestock agricultural industry, in partnership with the South African government, strategizes on a consistent farmer support plan of annual vaccination of the young animals using Smithburn vaccine (provided there are no cold chain issues). This will eliminate the chance of devastating outbreaks. If this were to become standard practice, it would improve and enhance the prospects of animal production to the advantage of South Africa's domestic and export markets and reduce the chance of a large-scale devastating outbreak event.

## Data Availability Statement

The original contributions presented in the study are included in the article/Supplementary Material, further inquiries can be directed to the corresponding author.

## Author Contributions

AA: conceptualization and writing—original draft. AA, RD, AK, JS, MKR, WB, RB, and CC: data collection and curation. AA and RD: formal analysis. WBK, MKR, and JTP: funding acquisition. AA, RD, JLS, WB, and MKR: methodology. RD, JLS, and WB: software. AA, RD, JLS, and WB: validation and visualization. All authors contributed to the article, writing-review and editing, and approved the submitted version.

## Funding

Understanding Rift Valley Fever in the Republic of South Africa, PI: WK. Defense Threat Reduction Agency—Biological Threat Reduction Program (DTRA-BTRP)—Thrust Area 6, CC WMD (HDTRA-14-1-0029). The project was sponsored by the US Department of Defense, Defense Threat Reduction Agency.

## Author Disclaimer

The content of the information does not necessarily reflect the position or the policy of the Federal government, and no official endorsement should be inferred.

## Conflict of Interest

JS is employed by Science Systems and Applications, Inc. The remaining authors declare that the research was conducted in the absence of any commercial or financial relationships that could be construed as a potential conflict of interest.

## Publisher's Note

All claims expressed in this article are solely those of the authors and do not necessarily represent those of their affiliated organizations, or those of the publisher, the editors and the reviewers. Any product that may be evaluated in this article, or claim that may be made by its manufacturer, is not guaranteed or endorsed by the publisher.
