# Supplementary material for: Climate Conditions During a Rift Valley Fever Post-epizootic Period in Free State, South Africa, 2014–2019
Source: Front Vet Sci. 2022 Jan 31;8:730424. doi: 10.3389/fvets.2021.730424 (PMC8848741; doi:10.3389/fvets.2021.730424)
Supplement: Supplementary file 1 [file Data_Sheet_1.pdf]

Supplementary Material

1 Supplementary Figures and Tables

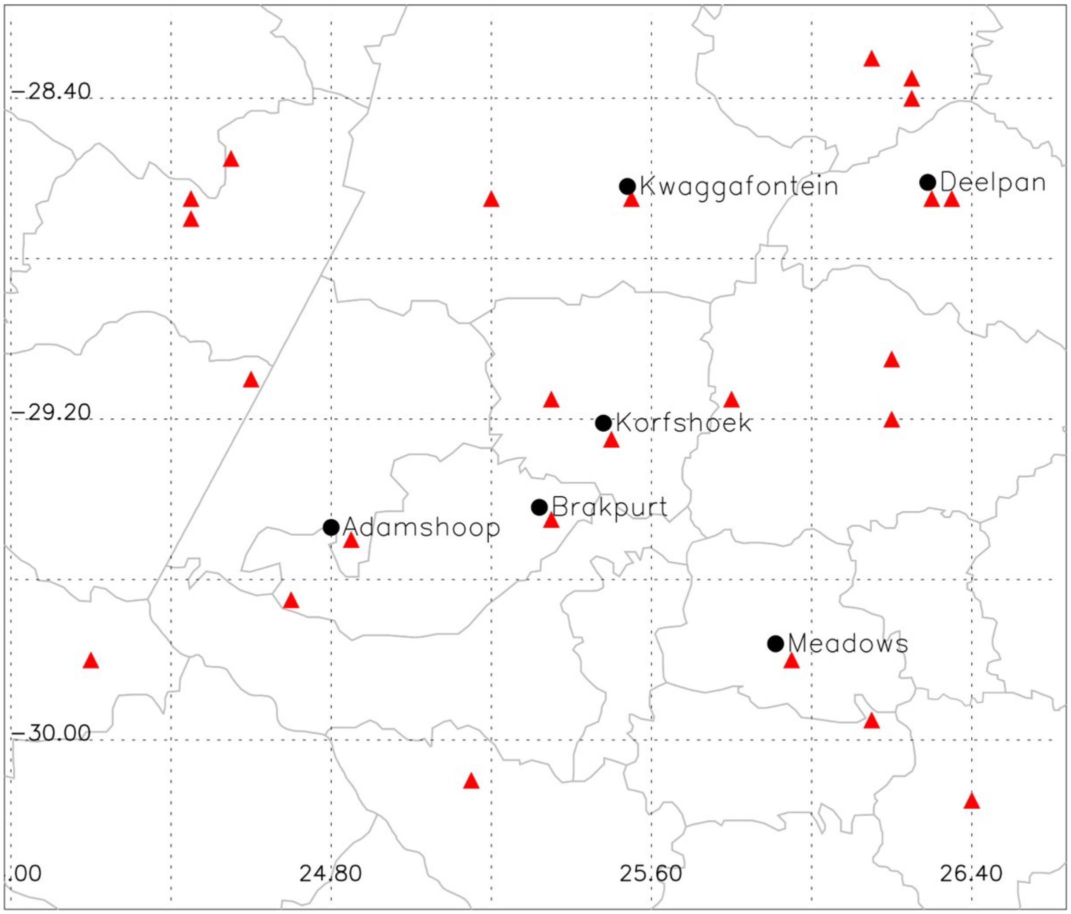

Supplementary Figure S1. Study area map showing mosquito vector sampling locations. Named locations correspond to selected sampling sites profiles in Figure 6 and Figure S3.

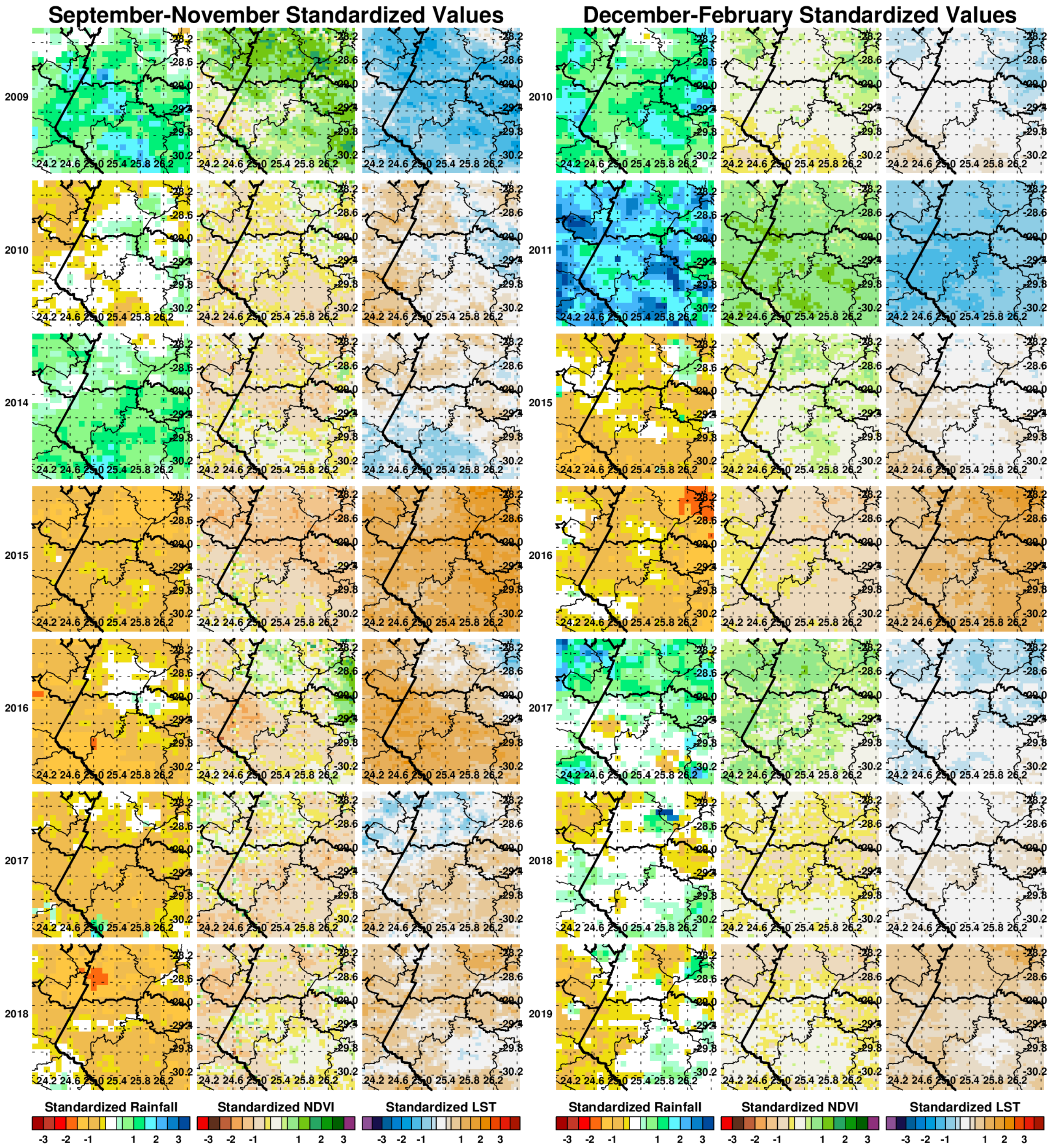
September-November Standardized Values
December-February Standardized Values
2009
2010
2014
2015
2016
2017
2018
2010
2011
2015
2016
2017
2018
2019
24.2 24.6 25.0 25.4 25.8 26.2
-28.2
-28.6
-29.0
-29.4
-29.8
-30.2
Standardized Rainfall
Standardized NDVI
Standardized LST
-3 -2 -1 1 2 3

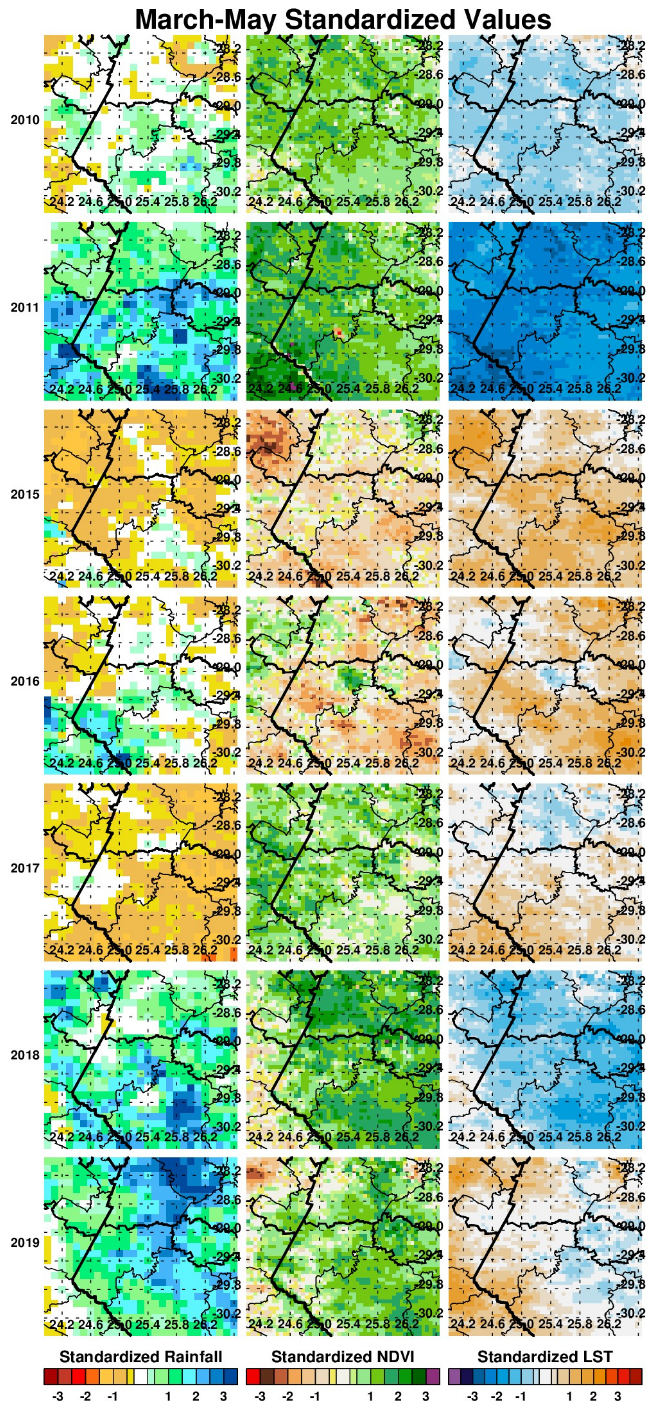

Supplementary Figure 2. Standardized seasonal anomaly patterns of rainfall, normalized difference vegetation index and land surface temperatures for epizootic seasons (2009/2010,2010/2011) and study/post-epizootic (interepizootic) period seasons from 2014-2019. Seasons are defined by early (September-November: SON), Mid (December – February: DJF) and end (March – May: MAM). Normalization/or standardization enables a straightforward comparison between the three climate metrics.

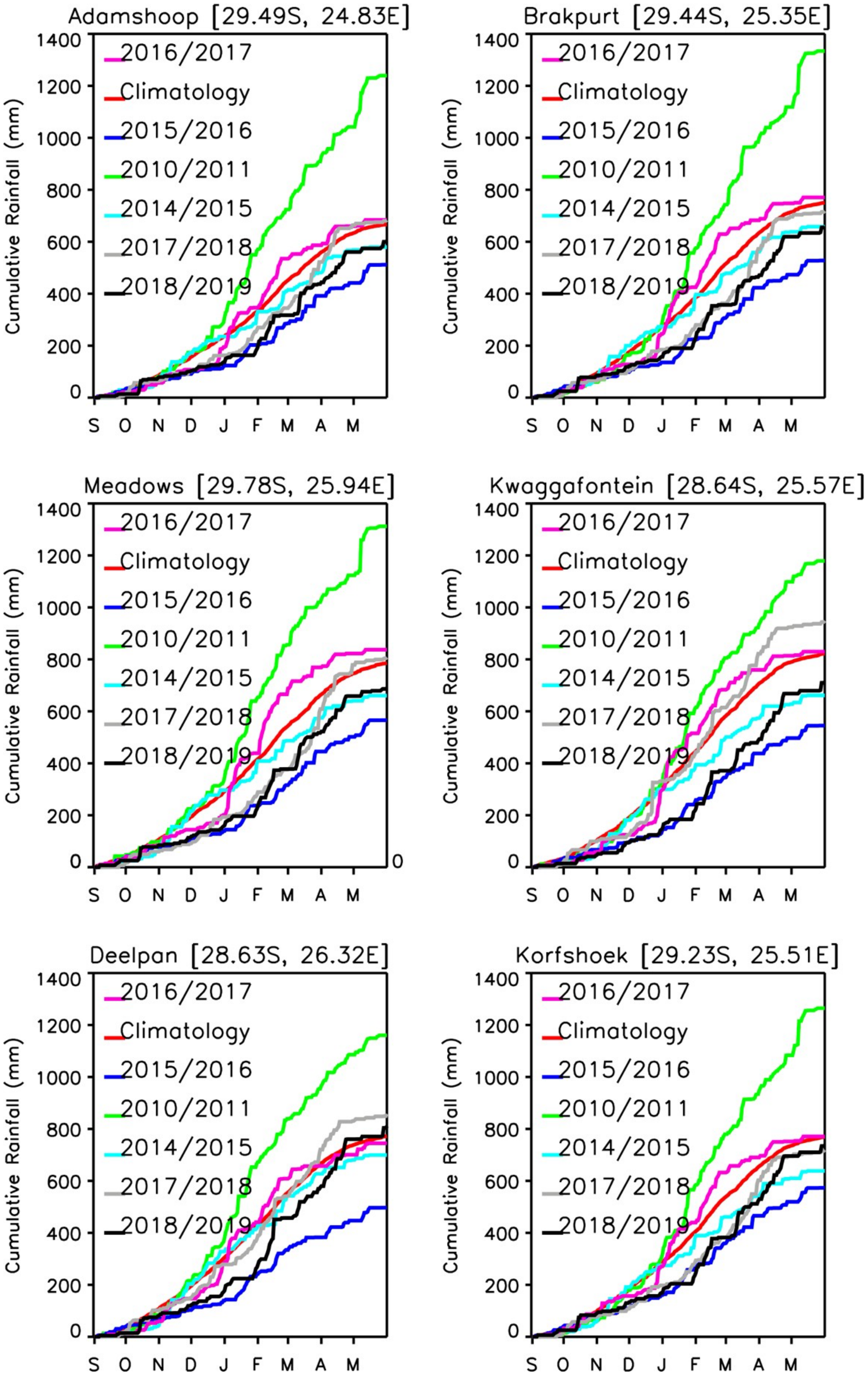
Adamshoop [29.49S, 24.83E]
Brakpurt [29.44S, 25.35E]
Meadows [29.78S, 25.94E]
Kwaggafontein [28.64S, 25.57E]
Deelpan [28.63S, 26.32E]
Korfshoek [29.23S, 25.51E]
Cumulative Rainfall (mm)
0
200
400
600
800
1000
1200
1400
S O N D J F M A M
2016/2017
Climatology
2015/2016
2010/2011
2014/2015
2017/2018
2018/2019

Supplementary Figure 3. Cumulative daily rainfall time series trajectories for various growing/mosquito seasons compared to the daily longterm mean rainfall (shown in red) for six selected study sites (shown in **Supplementary Figure S1)**. The epizootic season (2010-2011) shown in green, standout as way above the longterm mean, with an excess of ~300mm by April. Except for 2016/2017 and 2017/2018 seasons that are near normal or slightly above normal rainfall (later in the season), all the other seasons during the study/interepizootic period had below normal rainfall with a bottom out during the 2015/2016 (blue) season with a shortfall of ~200mm below the longterm mean by May.

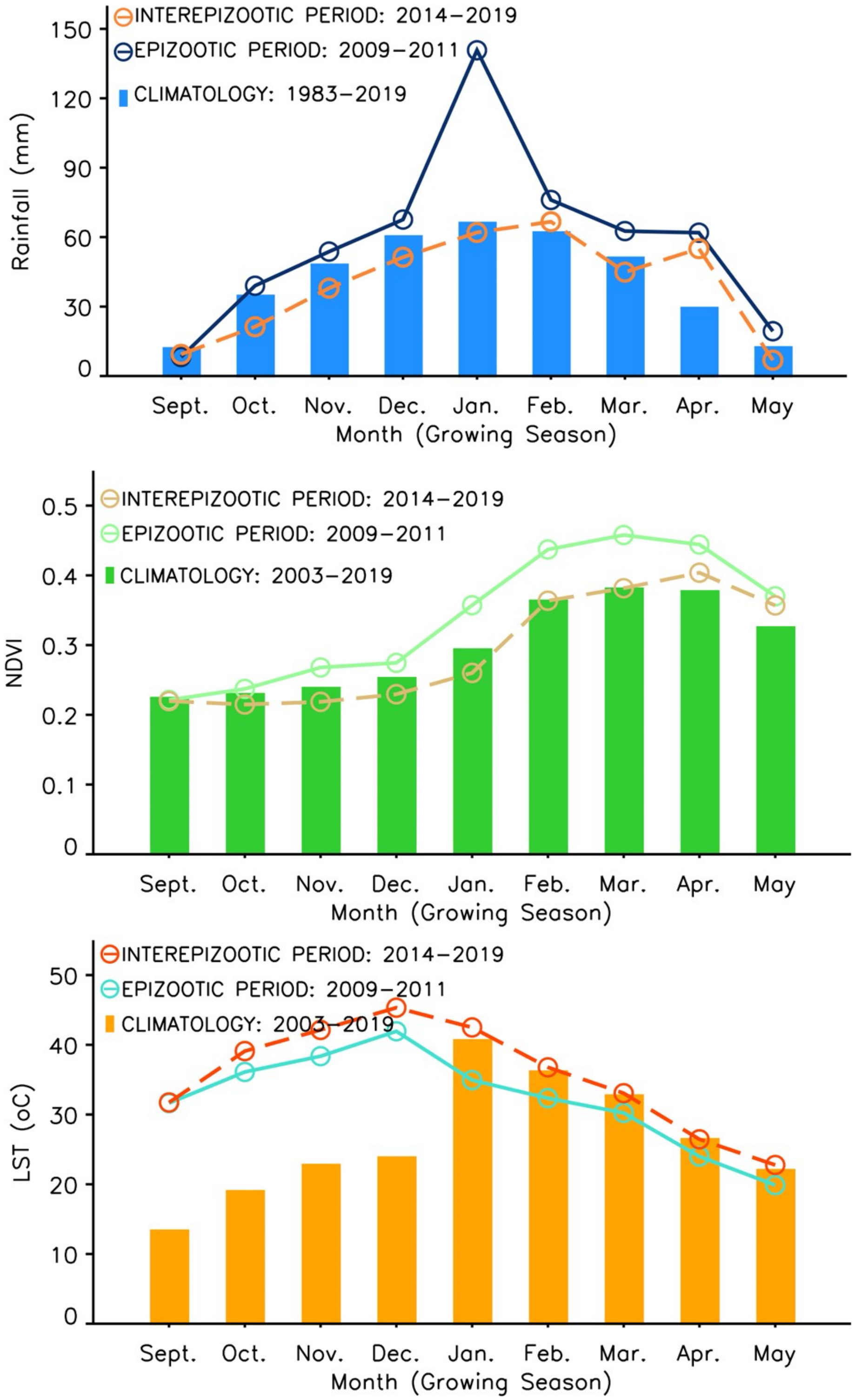

Supplementary Figure S4: Comparisons between epizootic (2010/11) and interepizootic (2014-2019) climate metric conditions for Rainfall (top), normalized difference vegetation index [NDVI] (middle) and land surface temperature [LST] (bottom).

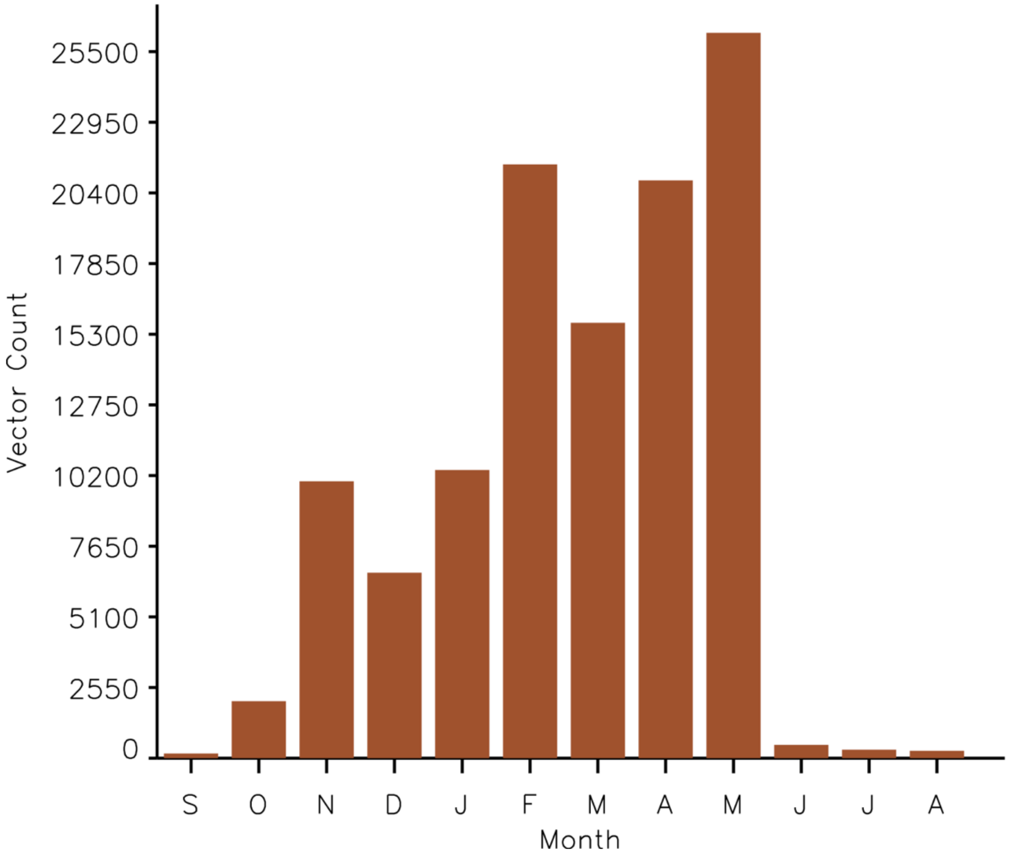

Supplementary Figure S5. Aggregate monthly distribution of vector populations collected during the study/interepizootic period. The trend shows that more numbers of vectors were collected towards the end of the season (April – May). This pattern agrees with the patterns in all the climate variables shown in Figure 3 and Figure S4.

Supplementary Table 1

Growing Season (September – May)	Rainfall			NDVI			LST		
	Max.	Min.	Average	Max.	Min.	Average	Max.	Min.	Average
**Epizootic period: 2009-2011**	148.28	1.46	58.80	0.50	0.21	0.34	44.87	17.50	32.15
**Inter-epizootic period2012-2019**	142.58	1.09	38.84	0.54	0.19	0.29	49.74	20.76	35.43
**Inter-epizootic /Study period 2014-2019**	142.58	1.09	39.47	0.54	0.19	0.29	49.74	20.76	35.53

Table S1: Area averaged [28S - 30.45S, 24E - 26.65E] growing season (September – May) absolute climate metrics (rainfall, normalized difference vegetation index[NDVI] and land surface temperature [LST]) comparison between the epizootic period (2009-2011) and the inter-epizootic period/and study period (2012-2019/2014-2019). Note that the metrics for the overlapping interepizootic/study period are the same indicating the persistence of low rainfall, reduced vegetation (NDVI) and warmer temperatures compared to the epizootic period.
